# Self-Reported Sensory Impairments and Changes in Cognitive Performance: A Longitudinal 6-Year Follow-Up Study of English Community-Dwelling Adults Aged ⩾50 Years

**DOI:** 10.1177/0898264318815391

**Published:** 2018-12-06

**Authors:** Ann E. M. Liljas, Kate Walters, Cesar de Oliveira, S. Goya Wannamethee, Sheena E. Ramsay, Livia A. Carvalho

**Affiliations:** 1 University College London, UK; 2 Newcastle University, Newcastle upon Tyne, UK; 3 Queen Mary University of London, UK

**Keywords:** aging, hearing loss, vision loss, dual sensory impairment, cognition

## Abstract

**Objective:** To investigate the influence of single and dual sensory impairments prospectively on cognition in adults aged ⩾50 years. **Method:** Community-dwelling English adults (*n* = 4,621) were followed up from 2008 to 2014. Self-reported hearing and vision were collected in 2008. Change in cognitive performance on working memory and executive function between 2008 and 2014 was evaluated. **Results:** Compared with good hearing and good vision, respectively, poor hearing and poor vision were associated with worse cognitive function (hearing: unstandardized coefficient *B* = 0.83, 95% Confidence Interval [CI] = [0.29, 1.37]; vision: *B* = 1.61, 95% CI = [0.92, 2.29] adjusted for age, sex, baseline cognition). Compared with no sensory impairment, dual sensory impairment was associated with worse cognition (*B* = 2.30, 95% CI = [1.21, 3.39] adjusted for age, sex, baseline cognition). All associations remained after further adjustment for sociodemographic characteristics, lifestyle factors, chronic conditions, falls, mobility, depression, and lack of companionship. **Discussion:** The findings are important as age-related sensory impairments are often preventable or modifiable, which may prevent or delay cognitive impairment.

## Introduction

Many populations worldwide including the population of England are aging due to increased life expectancy (Office for National Statistics, 2015; World Health Organization, 2015). Advanced age increases the risk of health problems including age-related loss of hearing and vision (Khaw, 1997). Hearing impairment is estimated to affect one in five (19%) adults aged 51 to 80 years in England and Wales (Akeroyd, Foreman, & Holman, 2014). Among older adults aged 60 years and above, 11% have a vision impairment (Royal National Institute of Blind People, 2013). Experiencing both hearing and vision impairment (dual sensory impairment) is estimated to affect at least 3% of the older population (Heine & Browning, 2015). The number of older adults affected by sensory impairments is, furthermore, likely to increase as the population ages (Gopinath et al., 2009; Helzner et al., 2005). Both hearing impairment and vision impairment have been associated with chronic diseases and disability (Crews & Campbell, 2004; Liljas et al., 2016a, 2016b, 2016c; West et al., 1997), age-related problems known for reducing the chances of good health, well-being, and independent living in later life (Campbell, Crews, Moriarty, Zack, & Blackman, 1999). This makes age-related sensory impairments an important public health concern. Another major health issue in later life is cognitive impairment, a key contributor to disability and dependence in older age (Lee et al., 2014; Mograbi, Faria, Fichman, Paradela, & Lourenco, 2014). The prevalence of cognitive impairment is increasing in England due to an aging population and increasing longevity (Office for National Statistics, 2016).

Several cross-sectional studies have shown associations of impairments in hearing and vision with cognitive impairment (Anstey, Lord, & Williams, 1997; F. R. Lin, 2011; F. R. Lin et al., 2011; Lindenberger & Baltes, 1994; Tay et al., 2006). There is also evidence from longitudinal studies reporting increased risks of incident cognitive impairment in those with hearing impairment after adjustment for sociodemographic characteristics and cardiovascular disease (CVD)-related measures (Fischer et al., 2016; F. R. Lin et al., 2013). However, other factors such as depression, social isolation, and mobility limitations were not considered in these studies. A previous study investigating impairments of hearing and vision with incident cognitive impairment found that hearing impairment and, in particular, vision impairment were associated with cognitive decline at 6-year follow-up (Valentijn et al., 2005). The results were, however, only adjusted for age, sex, and education. Other longitudinal studies have demonstrated that vision impairment, but not hearing impairment, was associated with an increased risk of incident cognitive impairment (Anstey, Luszcz, & Sanchez, 2001; M. Y. Lin et al., 2004), suggesting that vision impairment more than hearing impairment predicts cognitive decline. It has, furthermore, been suggested that the relationship between sensory impairments and subsequent cognitive impairment might not be unique to one sensory function (Fischer et al., 2016). There has been little research on dual sensory impairment and subsequent cognitive impairment (Heine & Browning, 2015). One study in women found a relationship between dual sensory impairment and incident cognitive impairment (M. Y. Lin et al., 2004), however, another study in both women and men did not observe an association between dual sensory impairment and cognitive decline (Hong, Mitchell, Burlutsky, Liew, & Wang, 2016). Therefore, this study, in a nationally representative cohort of English women and men aged ⩾50 years, aims to examine the influence of single and dual sensory functioning on cognitive function at 6-year follow-up on adjustment for a range of possible covariates, including baseline cognitive functioning.

## Method

### Study Design and Participants

**Figure 1.**
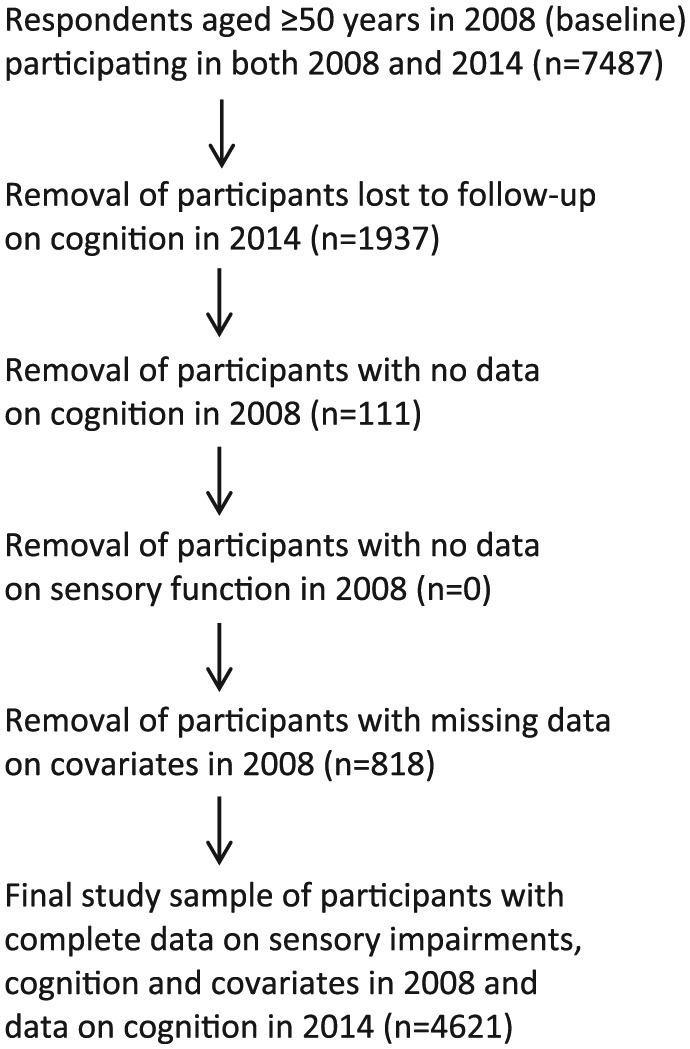
Derivation of the ELSA sample for this study. *Note.* ELSA = English Longitudinal Study of Ageing.

This study uses data from the English Longitudinal Study of Ageing (ELSA). ELSA is a prospective study of a nationally representative sample of men and women aged ⩾50 years who participated in the Health Survey for England in 1998, 1999, or 2001 (Marmot et al., 2015). Since 2002, participants have been followed up every 2 years for an interview on health and lifestyle and every 4 years for a physical examination. This study sample is restricted to the 4,621 participants (62% of respondents aged ⩾50 years in 2008) who undertook the cognitive tests in 2008 and 2014 and provided data on sensory function and covariates in 2008 (derivation of study sample outlined in Figure 1). All participants provided informed consent, and ethical approval for ELSA was obtained from the Multicentre Research and Ethics Committee.

### Hearing Impairment

Hearing function was assessed by asking participants to rate their hearing (using a hearing aid if they use one) as excellent, very good, good, fair, or poor. Reporting excellent, very good, or good hearing was classified as having good hearing, and this group formed the reference group. Experiencing fair or poor hearing was considered as poor hearing. The self-reported question used has previously been shown to be accurate when compared against objectively measured hearing (Ferrite, Santana, & Marshall, 2011; Gibson, Cronin, Kenny, & Setti, 2014).

### Vision Impairment

Vision function was measured using a validated self-reported question previously demonstrating a significant association with objectively measured eyesight, asking participants whether their eyesight was excellent, very good, good, fair, or poor using glasses or corrective lens if they normally do so (Zimdars, Nazroo, & Gjonca, 2012). Good vision was defined as reporting excellent, very good, or good eyesight and was used as the reference group. Reporting fair or poor eyesight was classified as poor vision.

### Dual Sensory Impairment

Being classified as having both poor hearing and poor vision was defined as having dual sensory impairment. Participants with no sensory impairment acted as the reference group because, as presented above, single sensory impairment is associated with adverse health outcomes. Having no impairment was considered as a more suitable reference to allow for understanding of the magnitude of the impact of having dual impairment compared with the general population.

### Assessment of Cognitive Performance

Participants’ cognitive status was first assessed in 2008 and then again in 2014, which allowed for participants to be followed up for 6 years. Cognition was based on a modified version of the cognitive score developed by Batty, Deary, and Zaninotto (2016) referring to working memory and executive function (Zaninotto & Batty, 2018). Working memory included immediate and delay recall tests of 10 nouns presented to the participants who were asked to recall as many words as possible immediately after the list was read, and then again after an approximately 5-min delay during which they completed other survey questions (range 0-20). Executive function was ascertained using a word-finding task asking participants to name as many different animals as possible in 1 min (range 0-60). In the original score, executive function also included a letter cancellation task, however, such data were not available in 2014. Similar to the calculations of the original score, the results from the three cognitive tests available were summed, providing a cognitive score (range 0-80), with a lower score indicating worse cognitive function. Change in cognitive function was calculated by subtracting baseline scores from the scores at follow-up.

### Covariates

Possible confounders considered in the analyses included age, sex, wealth, educational qualification, smoking, alcohol, physical activity, CVD, diabetes, and hypertension. Wealth was based on total net nonpension wealth (financial, housing, and physical wealth) of the household presented by quintiles. Educational qualification was defined as having an intermediate or higher qualification compared with no qualification. Smoking was defined as reporting being a current smoker or current nonsmoker. Alcohol consumption was based on frequency of consumption of all kinds of alcoholic drinks in the last 12 months and grouped into “daily,” “frequently” (once or twice a week or more, but not every day), “rarely” (once or twice a month/once every couple of months), and “never.” Physical activity was based on frequency and intensity in exercise by asking participants how often they engage in vigorous, moderate, and mild exercise (more than once a week, once a week, one to three times a month, hardly ever, or never). Participants who hardly ever or never engaged in vigorous, moderate, and mild activity were classified as sedentary. Engaging in mild activity one to three times a month, once a week, or more than once a week, or engaging in moderate activity one to three times a month was classified as low activity. Participants engaging in moderate activity once a week or more than once a week or vigorous activity one to three times a month were classified as being moderately active. Undertaking vigorous activity once a week or more than once a week was classified as high activity. Objective data on height and weight were used to calculate Body Mass Index (BMI). Self-reported doctor-diagnosed CVD (myocardial infarction, angina, and/or stroke), diabetes, and hypertension were analyzed dichotomously. Other important factors potentially associated with sensory impairments and cognitive impairment that were considered included history of falls, mobility limitations, depression, and lack of companionship. History of falls was based on participants reporting falling in the last 12 months. Mobility limitations referred to reporting problems walking 100 yards and/or climbing one flight of stairs. Depression was based on the validated 8-item version of the Center for Epidemiologic Studies Depression Scale (CES-D; Radloff, 1977). Scoring positively on 4 or more of the 8 items was classified as having depression. Feeling lack of companionship “some of the time” or “often” was combined and compared with feeling no lack of companionship.

### Statistical Analyses

Linear regression was used to assess longitudinal associations between impairments in hearing and vision (individually and combined) in 2008 with changes in the outcome variable cognition between 2008 and 2014. The regression models provided unstandardized coefficients *B* (the adjusted mean difference in the cognitive measure between those who did and those who did not report sensory impairments) with 95% Confidence Intervals (CI). Each domain of the cognitive score was, furthermore, tested individually. Sensory impairment (single/dual) was coded as 0, and no sensory impairment coded as 1. The statistical analyses were adjusted for age, sex, and cognitive function at baseline as well as covariates significantly associated with sensory impairments in this study sample (Table 1) and in previous research (Crews & Campbell, 2004; Liljas et al., 2016c). All variables were entered as categorical variables except for age and BMI, which were entered as continuous variables. All analyses were carried out using SPSS (Version 22, IBM, Armonk, New York).

**Table 1.** Age, Sex, Sociodemographic Characteristics, Lifestyle Factors, Comorbidities, Falls, Mobility Limitations, Depression, Lack of Companionship, and Cognitive Function by Hearing Function and Vision Function in a Cohort of 4,621 English Men and Women Aged 50 Years and Over in 2008 (Baseline).

	Overall	Good hearing	Poor hearing	*p* value	Good vision	Poor vision	*p* value
Totals, (*n*)%	4,621 (100)	3,761 (81)	860 (19)		4,143 (90)	478 (10)	
Covariates							
Age, *M* (*SD*)	64.9 (8.3)	64.4 (8.1)	67.1 (8.6)	<.01	64.7 (8.1)	67.3 (9.5)	<.01
Male gender, (*n*)%	2,100 (45)	1,590 (42)	510 (59)	<.01	1,906 (46)	194 (41)	.01
Wealth, (*n*)%							
1 (lowest)	636 (14)	487 (13)	149 (17)	.01	494 (12)	142 (30)	<.01
2	829 (18)	667 (18)	162 (19)		737 (18)	92 (19)	
3	935 (20)	761 (20)	174 (20)		843 (20)	92 (19)	
4	1,030 (22)	857 (23)	173 (20)		956 (23)	74 (16)	
5 (highest)	1,191 (26)	989 (26)	202 (24)		1,113 (27)	78 (16)	
No educational qualification, (*n*)%	1,015 (22)	787 (21)	228 (27)	<.01	857 (21)	158 (34)	<.01
Alcohol, (*n*)%							
Daily	763 (17)	604 (16)	159 (19)	.01	706 (17)	57 (12)	<.01
Frequently	2,223 (48)	1,845 (49)	378 (44)		2,015 (49)	208 (44)	
Rarely	859 (19)	707 (19)	152 (18)		770 (19)	89 (19)	
Never	776 (17)	605 (16)	171 (20)		652 (16)	124 (26)	
Smoker, (*n*)%	602 (13)	480 (13)	122 (14)	.14	508 (12)	94 (20)	<.01
Levels of physical activity, (*n*)%							
Sedentary	174 (4)	127 (3)	47 (6)	<.01	130 (3)	44 (9)	<.01
Low	568 (12)	433 (12)	135 (16)		459 (11)	109 (23)	
Moderate	2,327 (50)	1,895 (50)	432 (50)		2,086 (50)	241 (51)	
High	1,551 (34)	1,305 (35)	246 (29)		1,468 (35)	83 (17)	
BMI, *M* (*SD*)	28.3 (5.2)	28.3 (5.3)	28.4 (4.8)	.55	28.3 (5.2)	28.9 (5.6)	.01
Hypertension, (*n*)%	1,873 (41)	1,479 (39)	394 (46)	<.01	1,623 (39)	250 (52)	<.01
CVD, (*n*)%	538 (12)	390 (10)	148 (17)	<.01	441 (11)	97 (20)	<.01
Diabetes, (*n*)%	384 (8)	293 (8)	91 (11)	.01	322 (8)	62 (13)	<.01
History of falls, (*n*)%	819 (18)	615 (16)	204 (24)	<.01	682 (17)	137 (29)	<.01
Mobility limitations, (*n*)%	1,408 (31)	1,168 (28)	240 (50)	<.01	1,063 (28)	345 (40)	<.01
Depression, (*n*)%	540 (12)	421 (11)	119 (14)	.02	441 (11)	99 (21)	<.01
Lack of companionship, (*n*)%	1,603 (35)	1,296 (35)	307 (36)	.24	1,383 (34)	220 (47)	<.01
Outcomes							
Cognitive function, *M* (*SD*) in 2008	32.8 (8.3)	33.2 (8.2)	31.2 (8.5)	<.01	33.1 (8.1)	30.6 (9.1)	<.01
Cognitive function, *M* (*SD*) in 2014	31.4 (9.6)	32.0 (9.5)	29.1 (9.9)	<.01	31.8 (9.5)	27.9 (10.0)	<.01

*Note.* BMI = Body Mass Index; CVD = Cardiovascular Disease.

## Results

A total of 4,621 participants (55% women) aged ⩾50 years of a mean age of 64.9 years (*SD* 8.3) were included. One in five (19%) self-reported poor hearing, and 10% self-reported poor vision. Dual sensory impairment was prevalent in 179 participants (5% of 3,641 participants who had no sensory impairment or dual sensory impairment). On the cognitive scale ranging from 0 to 80, with higher scores demonstrating better cognitive function, average performance of all participants was 32.8 (*SD* 8.3) in 2008 and 31.4 (*SD* 9.6) in 2014.

Table 1 presents the characteristics of all participants in 2008 (baseline) for hearing impairment and vision impairment. Compared with participants with good hearing, those with poor hearing had significantly lower scores on cognitive function in both 2008 and 2014. Poor hearing was associated with being older, male, less wealthy, having no educational qualification, being less physically active, having chronic conditions including hypertension, CVD, and diabetes, a history of falls, mobility limitations, and depression. Participants with poor hearing were more likely to consume alcohol daily but also more likely to never drink, compared with participants with good hearing. Similarly, individuals with poor vision performed worse on cognition in 2008 and in 2014 than those with good vision. Poor vision was also associated with advanced age, being female, less wealth, no educational qualification, lower alcohol consumption, less physically active, BMI ⩾30, chronic conditions, falls, mobility limitations, depression, and lack of companionship. Table 2 shows the characteristics of 3,641 participants who had no sensory impairment (*n* = 3,462) or dual sensory impairment (*n* = 179). In comparison with participants with no sensory impairment, those with dual sensory impairment had lower scores on cognitive function in both 2008 and 2014 and were less wealthy, had no educational qualification, lower alcohol consumption, were more likely to smoke, less physically active, had chronic conditions, falls, mobility limitations, depression, and lack of companionship.

**Table 2.** Age, Sex, Sociodemographic Characteristics, Lifestyle Factors, Comorbidities, Falls, Mobility Limitations, Depression, Lack of Companionship, and Cognitive Function in a Cohort of 3,641 English Men and Women Aged 50 Years and Over With No Sensory Impairment Versus Dual Sensory Impairment in 2008 (Baseline).

	Overall	No sensory impairment	Dual sensory impairment	*p* value
Totals, (*n*)%	3,641 (100)	3,462 (95)	179 (5)	
Covariates				
Age, *M* (*SD*)	64.5 (8.1)	64.3 (7.9)	68.9 (9.5)	<.01
Male gender, (*n*)%	1,568 (43)	1,482 (43)	86 (48)	.17
Wealth, (*n*)%				
1 (lowest)	453 (12)	399 (12)	54 (30)	<.01
2	645 (18)	610 (18)	35 (20)	
3	737 (20)	703 (20)	34 (19)	
4	841 (23)	812 (24)	29 (16)	
5 (highest)	965 (27)	938 (27)	27 (15)	
No educational qualification, (*n*)%	767 (21)	698 (20)	69 (39)	<.01
Alcohol, (*n*)%				
Daily	589 (16)	568 (16)	21 (12)	<.01
Frequently	1,783 (49)	1,710 (49)	73 (41)	
Rarely	684 (19)	651 (19)	33 (18)	
Never	585 (16)	533 (15)	52 (29)	
Smoker, (*n*)%	452 (12)	419 (12)	33 (18)	.01
Levels of physical activity, (*n*)%				
Sedentary	123 (3)	103 (3)	20 (11)	<.01
Low	402 (11)	363 (11)	39 (22)	
Moderate	1,834 (50)	1,744 (50)	90 (50)	
High	1,282 (35)	1,252 (36)	30 (17)	
BMI, *M* (*SD*)	28.3 (5.3)	28.2 (5.3)	28.8 (5.3)	.18
Hypertension, (*n*)%	1,427 (39)	1,328 (38)	99 (55)	<.01
CVD, (*n*)%	379 (10)	336 (10)	43 (24)	<.01
Diabetes, (*n*)%	279 (8)	255 (7)	24 (13)	<.01
History of falls, (*n*)%	582 (16)	530 (15)	52 (29)	<.01
Mobility limitations, (*n*)%	1,033 (28)	928 (27)	105 (59)	<.01
Depression, (*n*)%	396 (11)	359(10)	37 (21)	<.01
Lack of companionship, (*n*)%	1,242 (34)	1,159 (34)	83 (47)	<.01
Outcomes				
Cognitive function, *M* (*SD*) in 2008	33.1 (8.2)	33.3 (8.1)	28.3 (8.8)	<.01
Cognitive function, *M* (*SD*) in 2014	31.9 (9.5)	32.2 (9.4)	25.5 (9.2)	<.01

*Note.* BMI = Body Mass Index; CVD = Cardiovascular Disease.

Table 3 presents the findings from the linear regression models investigating whether impairments in hearing and vision influence cognitive function at 6-year follow-up. The findings showed that both hearing impairment and vision impairment were associated with worse cognitive performance at 6-year follow-up (adjusted for age, sex, and cognitive function at baseline: hearing impairment unstandardized coefficient *B* = 0.83, 95% CI = [0.29, 1.37], *p* < .01; vision impairment unstandardized coefficient *B* = 1.61, 95% CI = [0.92, 2.29], *p* < .01). The associations remained after further adjustment for wealth, educational qualification, alcohol, smoking, physical activity, obesity, CVD, diabetes, hypertension, falls, mobility, depression, and lack of companionship with stronger associations observed for vision impairment (unstandardized coefficient *B* = 0.93, 95% CI = [0.22, 1.64], *p* = .01) than for hearing impairment (unstandardized coefficient *B* = 0.57, 95% CI = [0.03, 1.12], *p* = .04). Similarly, compared with participants with no sensory impairment, individuals with dual sensory impairment were more likely to demonstrate worse cognitive performance at 6-year follow-up (adjusted for age, sex, and cognitive function at baseline, unstandardized coefficient *B* = 2.30, 95% CI = [0.96, 3.13]) and the association remained after further adjustment for covariates (unstandardized coefficient *B* = 1.59, 95% CI = [0.36, 2.58]). As previous literature has suggested potential differences in outcomes between men and women with sensory impairments (Murphy & Gates, 1997; West et al., 1997), we tested for an interaction with gender, and this was nonsignificant.

**Table 3.** Unstandardized Coefficients *B* With 95% CI for Relationships of Vision Impairment, Hearing Impairment, and Dual Sensory Impairment at Baseline in 2008 With Changes in Cognitive Performance Between 2008 and 2014.

Hearing impairment and cognitive function (*n* = 4,621)	Unstandardized coefficient *B* (95% CI)	*p* value
M1: adjusted for age, sex, baseline cognitive function	0.83 [0.29, 1.37]	<.01
M2: M1 + wealth, education	0.73 [0.19, 1.27]	.01
M3: M2 + alcohol, smoking, physical activity, BMI	0.66 [0.12, 1.20]	.02
M4: M3 + CVD, diabetes, hypertension	0.64 [0.10, 1.18]	.02
M5: M4 + falls, mobility	0.59 [0.05, 1.14]	.03
M6: M5 + depression, lack of companionship	0.57 [0.03, 1.12]	.04
Vision impairment and cognitive function (*n* = 4,621)	Unstandardized coefficient *B* (95% CI)	*p* value
M1: adjusted for age, sex, baseline cognitive function	1.61 [0.92, 2.29]	<.01
M2: M1 + wealth, education	1.19 [0.49, 1.88]	<.01
M3: M2 + alcohol, smoking, physical activity, BMI	0.99 [0.29, 1.69]	.01
M4: M3 + CVD, diabetes, hypertension	0.96 [0.26, 1.66]	.01
M5: M4 + falls, mobility	0.94 [0.24, 1.64]	.01
M6: M5 + depression, lack of companionship	0.93 [0.22, 1.64]	.01
Dual sensory impairment and cognitive function (*n* = 3,641)	Unstandardized coefficient *B* (95% CI)	*p* value
M1: adjusted for age, sex, baseline cognitive function	2.30 [1.21, 3.39]	<.01
M2: M1 + wealth, education	1.86 [0.77, 2.95]	<.01
M3: M2 + alcohol, smoking, physical activity, BMI	1.67 [0.57, 2.76]	<.01
M4: M3 + CVD, diabetes, hypertension	1.64 [0.55, 2.74]	<.01
M5: M4 + falls, mobility	1.51 [0.41, 2.61]	.01
M6: M5 + depression, lack of companionship	1.59 [0.47, 2.71]	.01

*Note.* BMI = Body Mass Index; CI = Confidence Interval; M = Model; CVD = Cardiovascular Disease.

Supplementary analyses of each cognitive domain part of the cognitive score (Table S1) showed that poor hearing was associated with lower scores on immediate and delayed recall at 6-year follow-up. Poor hearing was not associated with lower scores on executive functioning at follow-up after adjustment for covariates. At 6-year follow-up, poor vision was associated with lower scores on executive functioning and immediate recall but not delayed recall after adjustment for covariates. Dual sensory impairment was associated with lower scores on all three cognitive domains at follow-up, and the associations remained after adjustment for covariates.

## Discussion

This study investigated the relationships of hearing impairment, vision impairment, and dual sensory impairment with change in cognitive performance at 6-year follow-up in English adults aged ⩾50 years. The results show that in this aging population, poor hearing, and poor vision, individually and combined, are associated with worse cognitive performance at 6-year follow-up. The associations observed remained after adjustment for a wide range of covariates including sociodemographic characteristics, lifestyle factors, chronic conditions, falls, mobility, depression, and lack of companionship.

Our study findings add to current literature on the relationships of impairments in hearing and vision with cognitive function as this is one of the very first studies examining dual sensory impairment and cognitive function longitudinally. Only two previous studies have investigated this relationship prospectively, reporting inconsistent findings; one study demonstrated an association between dual sensory impairment and incident risk of cognitive impairment at 4-year follow-up (M. Y. Lin et al., 2004). However, that study was in women only (*n* = 6,112). The other study did not observe an association between dual sensory impairment and cognitive decline at 5- and 10-year follow-up, possibly due to lack of statistical power, 93 (2.5%) of 3,654 participants reported dual sensory impairment at baseline; odds ratio (OR) = 1.41, 95% CI = [0.54, 3.72] and OR = 1.15, 95% CI = [0.28, 4.73], respectively (Hong et al., 2016). In our study, we demonstrated a relationship between dual sensory impairment and cognitive decline at 6-year follow-up in both women and men. The findings of the current study also contribute to existing literature on sensory impairments and subsequent cognitive impairment as it showed such relationships even after adjustment for covariates including falls, mobility, depression, and lack of companionship, factors not adjusted for in previous studies (Anstey et al., 2001; F. R. Lin, 2011; F. R. Lin et al., 2013; Lindenberger & Baltes, 1994; Tay et al., 2006; Valentijn et al., 2005). The dual effect on cognitive function appears to be additive, that is, above and beyond the presence of cognitive impairment alone, which is consistent with other evidence from the literature (Guthrie et al., 2018). It remains, however, unclear whether the relationship between sensory impairments and cognitive impairment is direct or indirect. A direct causal relationship might exist through poor sensory function reducing the opportunities to cognitive stimulation, leading to cognitive deterioration caused by cerebral atrophy (Lindenberger & Baltes, 1994). Alternatively, a direct causal relationship may be explained by poor sensory function requiring more cognitive resources to interpret information perceived, resulting in less cognitive capacity available for other cognitively demanding tasks (Baltes & Lindenberger, 1997). The associations observed between sensory impairments and worse cognitive performance on adjustment for a range of covariates support the hypotheses of a direct causal relationship.

The relationship between sensory impairments and worse cognitive performance could be due to shared age-related factors including degeneration of central nervous structures (Lindenberger & Baltes, 1994), or CVD (Crews & Campbell, 2004; Dregan, Stewart, & Gulliford, 2013). While our study showed a relationship of sensory impairments with worse cognitive performance after adjustment for CVD and CVD-related conditions such as hypertension, diabetes, and CVD risk factors including smoking, a higher BMI, and physical activity, there may be residual (unmeasured) confounding. There could also be psychosocial factors such as depression and social isolation linking vision impairment to poor future cognitive performance (Barnes, Mendes de Leon, Wilson, Bienias, & Evans, 2004; Heine & Browning, 2004). In our study, we further explored the independent effect of sensory impairments on cognitive performance after adjustment for the psychosocial factors of depression and lack of companionship. However, the measures available with sufficient data may have incompletely captured these domains. Other aspects including anxiety, participation in social activities, and subjective feelings of loneliness may also be important. It is also possible that the relationship is due to underlying mechanisms such as inflammation (Peracino & Pecorelli, 2016).

### Strengths and Limitations

The major strengths of this study are that it is based on data of older English adults from a large population-based cohort. The participants were followed up 6 years later for changes in cognitive function, and the models were adjusted for a wide range of potential covariates.

Limitations include that hearing impairment and vision impairment were self-reported rather than objectively measured. However, the questions used have been validated against objective measures (Ferrite et al., 2011; Gibson et al., 2014; Zimdars et al., 2012), and the prevalence rates of sensory impairments reported are similar to national estimates (Akeroyd et al., 2014; Royal National Institute of Blind People, 2013). Sensory function was assessed at baseline only and data on the primary cause of and change in sensory function were not available. Also, data on type of and frequency of use of glasses/lenses and hearing aids were not available. Furthermore, the differences in cognitive performance associated with sensory impairments were fairly small and may not be clinically relevant.

A modified version rather than the original cognitive score by Batty et al. (2016) was used. While the original cognitive score included three domains of cognitive function—working memory, executive function, and processing speed—data on processing speed were not collected in 2014 and, hence, not available for the analyses conducted in this study. The working memory tests asking the participants to recall 10 common nouns required some degree of hearing to complete. Miscommunication was minimized by verbal information being provided face-to-face in a quiet environment by experienced examiners accustomed to working with older adults. The list of words used for the memory tests was furthermore presented by a recorded computer voice, and the volume was adjusted prior to the test if necessary (Marmot et al., 2015). Nevertheless, supplementary analyses of the individual cognitive domains showed that poor hearing was associated with immediate and delayed recall (domains requiring adequate hearing) but not with executive functioning (no hearing required), and difficulty in initial hearing of the words may have impacted on their performance in the tests of recall. However, no potential study participants reported being unable to undertake the recall tests due to deafness. The measurements of cognition (naming animals and recall of words) did not require adequate eyesight. The exclusion of participants who had incomplete data on sensory impairments, cognition, and covariates raises the possibility of a selection bias toward healthier participants. In keeping with most longitudinal cohort studies, we observed that, indeed, 1,937 participants with baseline measures eligible for participation in the study lost to follow-up were more likely to be older (*p* < .01), less wealthy (*p* < .01), and in poorer health, including more likely to be a current smoker (*p* < .01) and having CVD (*p* < .01), depression (*p* < .01), and mobility limitations (*p* < .01). Thus, the associations between sensory impairments and worse cognitive function observed in our study sample of a “younger” and “healthier” population with complete data might have been even stronger in a sample that included the nonrespondents, too. Study limitations also include several unmeasured and incompletely addressed factors of potential importance (e.g., anxiety and low social engagement) that may have confounded the relationship of impairments in hearing and/or vision and cognitive decline. The study was furthermore carried out in a population of “younger old” adults (average 64.9 years) predominantly of White English ethnic origin. It may, therefore, not be appropriate to extrapolate our findings to other older populations.

## Conclusion

In our study, aging adults with individual and combined impairments in hearing and vision had greater risks of worse cognitive performance at 6-year follow-up compared with those with good sensory function. Sensory impairments can often be prevented or modified and targeting sensory impairments in aging adults could have potential to prevent or delay cognitive impairment. This is of importance to reduce the risk of cognitive impairment, a key contributor to disability, dependency, and mortality in England.

## Supplemental Material

Supplementary_table_1_AL220518 – Supplemental material for Self-Reported Sensory Impairments and Changes in Cognitive Performance: A Longitudinal 6-Year Follow-Up Study of English Community-Dwelling Adults Aged ⩾50 Years Click here for additional data file. Supplemental material, Supplementary_table_1_AL220518 for Self-Reported Sensory Impairments and Changes in Cognitive Performance: A Longitudinal 6-Year Follow-Up Study of English Community-Dwelling Adults Aged ⩾50 Years by Ann E. M. Liljas, Kate Walters, Cesar de Oliveira, S. Goya Wannamethee, Sheena E. Ramsay and Livia A. Carvalho in Journal of Aging and Health
