# Supplementary material for: Self-Reported Sensory Impairments and Changes in Cognitive Performance: A Longitudinal 6-Year Follow-Up Study of English Community-Dwelling Adults Aged ⩾50 Years
Source: J Aging Health. 2018 Dec 6;32(5-6):243–51. doi: 10.1177/0898264318815391 (PMC7221867; doi:10.1177/0898264318815391)
Supplement: Supplementary_table_1_AL220518 – Supplemental material for Self-Reported Sensory Impairments and Changes in Cognitive Performance: A Longitudinal 6-Year Follow-Up Study of English Community-Dwelling Adults Aged ⩾50 Years [file Supplementary_table_1_AL220518.pdf]

Supplementary table 1. Beta coefficients with 95% confidence intervals (CI) for relationships of vision impairment, hearing impairment and dual sensory impairment at baseline in 2008 with each of the three cognitive domains; executive functioning, immediate recall of 10 words, and delayed recall of 10 words at 6 years of follow up in 2014

	Poor hearing		Poor vision		Dual sensory impairment	
**Cognitive domain**	n=4621		n=4621		n=3641	
**Executive functioning (animal naming)**	**β (95% CI)**	**p-value**	**β (95% CI)**	**p-value**	**β (95% CI)**	**p-value**
Model 1 (M1): adjusted for age, sex, executive functioning at baseline	0.45 (0.01, 0.88)	0.03	1.06 (0.51, 1.61)	<0.01	1.60 (0.72, 2.49)	<0.01
Model 2 (M2): M1 + wealth, education	0.35 (-0.08, 0.79)	0.11	0.74 (0.18, 1.30)	0.01	1.26 (0.38, 2.15)	0.01
Model 3 (M3): M2 + alcohol, smoking, physical activity, BMI	0.30 (-0.13, 0.74)	0.17	0.60 (0.04, 1.16)	0.04	1.11 (0.22, 2.00)	0.02
Model 4 (M4): M3 + CVD, diabetes, hypertension	0.29 (-0.15, 0.73)	0.19	0.60 (0.03, 1.16)	0.04	1.10 (0.21, 1.99)	0.02
Model 5 (M5): M4 + falls, mobility	0.27 (-0.17, 0.70)	0.24	0.57 (0.01, 1.14)	0.05	1.02 (0.13, 1.92)	0.03
Model 6 (M6): M5 + depression, lack of companionship	0.25 (-0.20, 0.69)	0.27	0.58 (0.01, 1.16)	0.05	1.08 (0.16, 1.99)	0.02
**Immediate recall of 10 words**						
Model 1 (M1): adjusted for age, sex, immediate recall at baseline	0.27 (0.15, 0.38)	<0.01	0.42 (0.28, 0.57)	<0.01	0.61 (0.38, 0.84)	<0.01
Model 2 (M2): M1 + wealth, education	0.23 (0.12, 0.35)	<0.01	0.31 (0.16, 0.46)	<0.01	0.47 (0.24, 0.69)	<0.01
Model 3 (M3): M2 + alcohol, smoking, physical activity, BMI	0.22 (0.10, 0.33)	<0.01	0.27 (0.12, 0.41)	<0.01	0.43 (0.20, 0.66)	<0.01
Model 4 (M4): M3 + CVD, diabetes, hypertension	0.21 (0.09, 0.31)	<0.01	0.26 (0.11, 0.40)	<0.01	0.41 (0.18, 0.63)	<0.01
Model 5 (M5): M4 + falls, mobility	0.20 (0.08, 0.31)	<0.01	0.24 (0.09, 0.39)	<0.01	0.38 (0.15, 0.61)	<0.01
Model 6 (M6): M5 + depression, lack of companionship	0.20 (0.08, 0.32)	<0.01	0.23 (0.08, 0.38)	<0.01	0.38 (0.15, 0.62)	<0.01
**Delayed recall of 10 words**						
Model 1 (M1): adjusted for age, sex, delayed recall at baseline	0.27 (0.14, 0.40)	<0.01	0.33 (0.17, 0.49)	<0.01	0.50 (0.25, 0.76)	<0.01
Model 2 (M2): M1 + wealth, education	0.25 (0.12, 0.38)	<0.01	0.22 (0.05, 0.39)	0.01	0.37 (0.11, 0.63)	0.01
Model 3 (M3): M2 + alcohol, smoking, physical activity, BMI	0.23 (0.10, 0.36)	<0.01	0.17 (0.01, 0.34)	0.04	0.33 (0.07, 0.59)	0.01
Model 4 (M4): M3 + CVD, diabetes, hypertension	0.22 (0.09, 0.35)	<0.01	0.17 (0.00, 0.33)	0.05	0.32 (0.06, 0.58)	0.02
Model 5 (M5): M4 + falls, mobility	0.21 (0.08, 0.34)	<0.01	0.15 (-0.02, 0.32)	0.07	0.29 (0.03, 0.55)	0.03
Model 6 (M6): M5 + depression, lack of companionship	0.21 (0.08, 0.34)	<0.01	0.14 (-0.03, 0.31)	0.10	0.30 (0.03, 0.57)	0.03
